# Comparison of four *phaC *genes from *Haloferax mediterranei *and their function in different PHBV copolymer biosyntheses in *Haloarcula hispanica*

**DOI:** 10.1186/1746-1448-6-9

**Published:** 2010-08-20

**Authors:** Jing Han, Ming Li, Jing Hou, Linping Wu, Jian Zhou, Hua Xiang

**Affiliations:** 1 State Key Laboratory of Microbial Resources, Institute of Microbiology, Chinese Academy of Sciences, No.1 West Beichen Road, Chaoyang District, Beijing 100101, China; 2 Multidisciplinary Research Center, Shantou University, China

## Abstract

**Background:**

The halophilic archaeon *Haloferax mediterranei *is able to accumulate large amounts of poly(3-hydroxybutyrate-*co*-3-hydroxyvalerate) (PHBV) with high molar fraction of 3-hydroxyvalerate (3HV) from unrelated carbon sources. A Polyhydroxyalkanoate (PHA) synthase composed of two subunits, PhaC_Hme _and PhaE_Hme_, has been identified in this strain, and shown to account for the PHBV biosynthesis.

**Results:**

With the aid of the genome sequence of *Hfx. mediterranei *CGMCC 1.2087, three additional *phaC *genes (designated *phaC1*, *phaC2*, and *phaC3*) were identified, which encoded putative PhaCs. Like PhaC_Hme _(54.8 kDa), PhaC1 (49.7 kDa) and PhaC3 (62.5 kDa) possessed the conserved motifs of type III PHA synthase, which was not observed in PhaC2 (40.4 kDa). Furthermore, the longer C terminus found in the other three PhaCs was also absent in PhaC2. Reverse transcription PCR (RT-PCR) revealed that, among the four genes, only *phaC*_Hme _was transcribed under PHA-accumulating conditions in the wild-type strain. However, heterologous coexpression of *phaE*_Hme _with each *phaC *gene in *Haloarcula hispanica *PHB-1 showed that all PhaCs, except PhaC2, could lead to PHBV accumulation with various 3HV fractions. The three kinds of copolymers were characterized using gel-permeation chromatography (GPC), differential scanning calorimetry (DSC), and thermogravimetric analysis (TGA). Their thermal properties changed with the variations in monomer composition as well as the different molecular weights (*M*_w_), thus might meet various application requirements.

**Conclusion:**

We discover three cryptic *phaC *genes in *Hfx. mediterranei*, and demonstrate that genetic engineering of these newly identified *phaC *genes has biotechnological potential for PHBV production with tailor-made material properties.

## Background

Polyhydroxyalkanoates (PHAs) are polymers of hydroxyalkanoates and are synthesized by various bacteria and some haloarchaeal strains [1]. The molecular weight of PHAs is generally distributed in the range of 50,000-1,000,000 Da, which is sufficient to provide these polymers with similar characteristics as conventional plastics [2]. However, due to the high production cost of PHAs, semi-commercial production has been realized only for the two common polyesters, poly(3-hydroxybutyrate) (PHB) and poly(3-hydroxybutyrate-*co*-3-hydroxyvalerate) (PHBV) [3-5]. Recently, halophilic Archaea have been attracting strong interest in PHBV research due to their comparable advantages as PHA producers, including utilizing unrelated cheap carbon sources, non-strict sterilization and easy method for PHA extraction [6-8]. A few haloarchaeal strains have been found to synthesize intracellular short-chain-length PHAs (scl-PHAs), PHB and PHBV, to serve as carbon and energy storage [8-16]. Compared with PHB, PHBV possesses better polymer properties and thus has wider applications in commodity materials [17-19]. Among the halophilic archaea, *Haloferax mediterranei *is one of the best-studied strains due to its capability to accumulate large amounts of PHBV from inexpensive carbon sources, including glucose, starch, and whey [6-8,20,21]. Therefore, it has been evaluated as one of the most promising candidate prokaryotes for the industrial production of PHBV [7].

PHA synthases are the key enzymes of PHA synthesis and catalyze the incorporation of 3-hydroxyacyl-CoA into PHAs. Over the past few decades, extensive research has been devoted to the study of PHA synthases in the domain of Bacteria. This enzyme is grouped into four types in bacteria, according to its substrate specificity and subunit composition [22]. The typical bacterial type III synthase is composed of two subunits, PhaE (~40 kDa) and PhaC (~40 kDa) [23]. In the domain of Archaea, a novel PHA synthase homologous to the bacterial type III PHA synthase, with subunits designated PhaC_Hme _and PhaE_Hme_, has been identified and characterized in *Hfx. mediterranei *[6]. As in bacteria, the PHA synthase-encoding genes (*phaEC*_Hme_) in *Hfx. mediterranei *are clustered and co-transcribed, and both PhaC_Hme _and PhaE_Hme _are indispensable for the PHBV synthesis from multiple unrelated carbon sources. The haloarchaeal PHA synthase and bacterial type III synthase share some conserved residues (e.g., the Cys-Asp-His catalytic triad). However, they are quite different in molecular weights and several characteristic motifs. The PhaC_Hme _(54.8 kDa) is much larger than its bacterial counterparts, in which the longer sequence at the C terminus has been shown to be indispensable for enzyme activity [6]. In contrast, the PhaE_Hme _(~20 kDa) subunit is much smaller than its bacterial counterparts. Moreover, although "PhaE box" is strongly conserved in PhaEs of bacteria, it is missing in PhaE_Hme _of *Hfx. mediterranei*.

Recently, the genome sequence of *Hfx. mediterranei *CGMCC 1.2087 has been completed in our laboratory (Han J. *et al*: Bioplastic production in the domain of Archaea: insights from the genome sequence of *Haloferax mediterranei*, in preparation). Intriguingly, sequence analysis revealed that three additional *phaC *paralogs (named *phaC1*, *phaC2*, and *phaC3*) were present and scattered throughout the whole genome. In this study, we compared the features of PhaC_Hme _and these three putative PhaC proteins (PhaC1-PhaC3), and determined their capabilities of PHA polymerizing in *Haloarcula hispanica*. Furthermore, the material properties of the produced polymers were also characterized using multiple methods.

## Results

### Identification and analysis of the *phaC* genes in *Hfx. mediterranei*

*Hfx. mediterranei *is comprised of one chromosome and three extrachromosomal elements (megaplasmids) [24]. The genome sequence of *Hfx. mediterranei *CGMCC 1.2087 has been recently completed by our group. The genes encoding its PHA synthase, named *phaE*_Hme _and *phaC*_Hme_, have already been cloned and characterized [6] and were found to be located on megaplasmid pHM300. Interestingly, analysis of the whole genome sequence revealed that three other *phaC *paralogs existed in this strain and were designated *phaC1*, *phaC2*, and *phaC3*.

**Figure 1:**
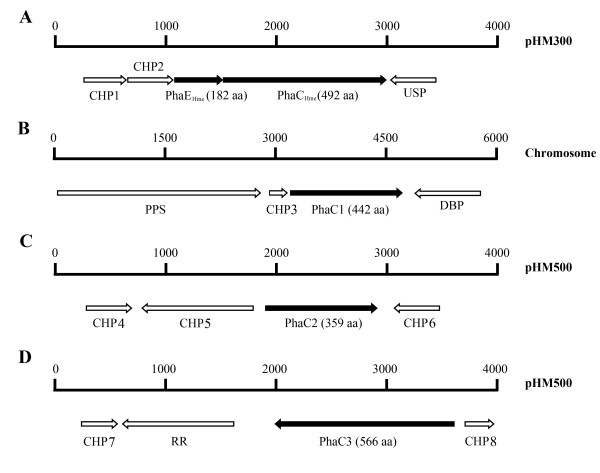
**Schematic overview of the four *phaC *genes with their adjacent genes in *Hfx. mediterranei***. A: PhaEC_Hme _encoded by pHM300; B: PhaC1 encoded by the chromosome; C: PhaC2 encoded by pHM500; D: PhaC3 encoded by pHM500. The genes are shown as arrows. The numeric scale above the genes gives the length of the DNA (bp). The PHA synthase-encoding genes are shown in black. CHP: conserved hypothetical protein; DBP: DNA binding protein; PPS: phosphoenolpyruvate synthase; RR: Response regulator; USP: universal stress protein.

**Figure 2:**
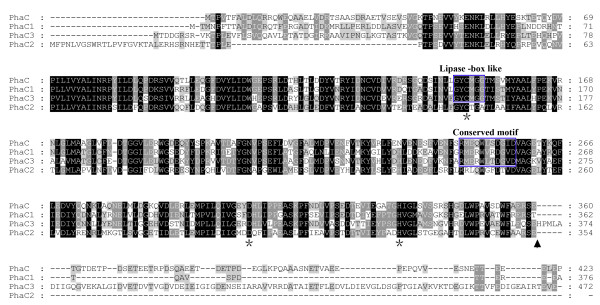
**Multiple alignments of the amino acid sequences of four PhaCs from *Hfx. mediterranei***. The "Lipase box-like" and highly conserved motif of the Type III synthase are indicated. The conserved catalytic triad residues are indicated with asterisks. The position indicated with an arrow represents the C terminus sequence missing in PhaC2. Amino acids are given in standard one-letter abbreviations and the numbers indicate the positions of the amino acids within the respective proteins. Shaded amino acids residues indicate identical and conserved residues in the sequences of the four PhaCs.

The *phaC1 *was mapped to the chromosome, whereas *phaC2 *and *phaC3 *were both mapped to megaplasmid pHM500 (Figure 1). Most of the genes adjacent to the four *phaC*s encoded conserved hypothetical proteins with unknown functions (Figure 1). The four PhaC proteins of *Hfx. mediterranei *exhibited homology to each other (43%-58% identity), and they all contained the catalytic triad residues (Cys-Asp-His) (Figure 2). Both the "Lipase box-like" sequence (Gly-X-Cys-X-Gly-Gly) and the conserved motif of type III PHA synthase were present in PhaC_Hme_, PhaC1, and PhaC3 (Figure 2). In the case of PhaC2, the last "Gly" residue of the "Lipase box-like" sequence was replaced by an "Ala", and the conserved motif of the type III PHA synthase was also not strongly conserved (Figure 2). The deduced molecular weights of the four PhaCs were as follows: PhaC_Hme_-54.8 kDa, PhaC1-49.7 kDa, PhaC2-40.4 kDa, and PhaC3-62.5 kDa. Only the molecular weight of PhaC_Hme _was equivalent to that of the PhaCs from *Haloarcula marismortui *(53.1 kDa; Accession no., YP_137339), *Haloarcula hispanica *(53.0 kDa; Accession no., ABV71394), and *Haloquadratum walsbyi *(52.1 kDa, Accession no., YP_658052). When compared with the primary sequences of the other three PhaCs, the C terminal sequence was missing in PhaC2, which resulted in the smallest molecular weight.

### Only *phaC*_*Hme*_was transcribed in *Hfx. mediterranei* in laboratory conditions

**Figure 3:**
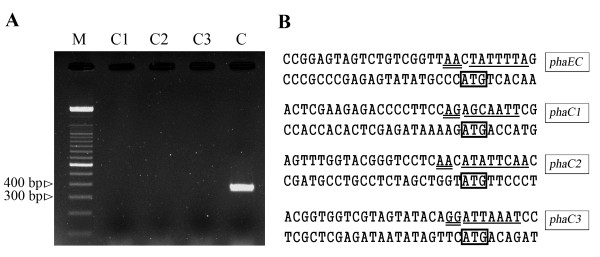
**Transcriptional analysis of four *phaC *genes**. (A) RT-PCR products of four *phaC *genes. Lane C1: *phaC1 *(391 bp); lane C2: *phaC2 *(408 bp); lane C3: *phaC3 *(412 bp); lane C: *phaC*_Hme _(382 bp); lane M: 100 bp DNA marker. (B) Promoter regions of the *phaC *genes. The putative TATA-box (single underlined), BRE (double underlined), and the translational start codons (boxed) of *phaE*_Hme_, *phaC1*, *phaC2*, and *phaC3 *are indicated.

In previous work, we found that the *phaEC*_Hme_-deleted strain *Hfx. mediterranei *PHBV-1 could not accumulate detectable scl-PHAs and expression of *phaE*_Hme _alone could not restore the ability to accumulate scl-PHAs [6], which indicated that there were no additional functional PhaC subunits in *Hfx. mediterranei *PHBV-1. To provide some clues to this phenomenon, RT-PCR with gene-specific primer pairs for the four *phaC *genes (Table 1) was employed to determine whether they were transcribed in the wild-type strain. The lengths of the PCR products of *phaC*_Hme_, *phaC1*, *phaC2*, and *phaC3 *were 382 bp, 391 bp, 408 bp, and 412 bp, respectively. The results demonstrated that of the four genes, only *phaC*_Hme _was actively transcribed in MG medium (Figure 3A), which was consistent with our previous observation that PhaC_Hme _was indispensable for PHBV biosynthesis in *Hfx. mediterranei*, as the other three *phaCs *were not expressed.

**Table 1:** Plasmids and primers used in this study.

Plasmids or primers	Relevant characteristics	Sources or references
**Plasmids**		
pWL3E	11.2-kb, *phaE*_Hme _and its native promoter	[6]
pWL3EC	12.6-kb, *phaEC*_Hme _and its native promoter	[6]
pWL3EC1	12.5-kb, derivative of pWL3E, *phaC1 *insert	This study
pWL3EC2	12.2-kb, derivative of pWL3E, *phaC2 *insert	This study
pWL3EC3	12.9-kb, derivative of pWL3E, *phaC3 *insert	This study

**Primers for RT-PCR (5'-3' sequence)**		
phaC-RT-F	GTACATTCTCGACCTCCA	
phaC-RT-R	AGGAACGTTTCCAAACGC	
phaC1-RT-F	ATGACCATGAACCCGTTT	
phaC1-RT-R	CGATGTACCGATTCACGT	
phaC2-RT-F	ATGTTCCCTAACCTCGTC	
phaC2-RT-R	GTCCGCCTCAGTTCGTTC	
phaC3-RT-F	ATGACAGATGATGGGCGT	
phaC3-RT-R	CGATGTAGCGATTGACGT	
**Primers for expression (5'-3' sequence **^**a**^**)**		
phaC1-BamHI-F	ATAGGATCCATGACCATGAACCCGTTT	
phaC1-KpnI-R	AGCGGTACCTTAGCTGGTGCGTTGTCT	
phaC2-BamHI-F	ATAGGATCCATGTTCCCTAACCTCGTC	
phaC2-KpnI-R	AGCGGTACCTTATCCGTTTGGTGACGC	
phaC3-BamHI-F	ATAGGATCCATGACAGATGATGGGCGT	
phaC3-KpnI-R	AGCGGTACCTCAGTCATCTTCTGTACC	

^a^Sequences representing the restriction sites are underlined.

### Genetic analysis of the functions of the four *PhaCs* in *Haloarcula*

To determine if *phaC1*, *phaC2*, and *phaC3 *could encode functional proteins, a PHA synthase gene-deleted strain, named *Har. hispanica *PHB-1 [13], was used to heterologously express these proteins. Because PhaC proteins were proposed to be functional combined with the PhaE subunit, the three expression plasmids designated pWL3EC1, pWL3EC2, and pWL3EC3 (Table 1) were constructed based on the PhaE_Hme_-expression plasmid pWL3E [6] and individually introduced into *Har. hispanica *PHB-1. During fermentation, *Har. hispanica *PHB-1/pWL3E [6] and the wild-type strain were used as the negative and positive control, respectively. To compare the PHBV synthesized by the four PHA synthases with distinct PhaC subunits, *Har. hispanica *PHB-1/pWL3EC [6] harboring the *phaEC*_Hme _genes of *Hfx. mediterranei *was also employed.

Table 2 summarized the results of PHA accumulation in *Har. hispanica *and its five transformants. Compared with the wild-type strain, there was no significant difference in final cell yield as evaluated by cell dry weight (CDW). The expression of PhaE_Hme _alone or coexpression with PhaC2 did not lead to the synthesis of any PHA. However, the other three PhaC proteins (PhaC_Hme_, PhaC1 and PhaC2) were functional during PHBV polymerization when excess glucose was supplied. PHBV content accumulated in recombinant PHB-1 strains harboring PhaEC_Hme _(17.33%) or PhaEC1 (15.61%) was a little higher than that of wild type (12.26%), and resulted in a much higher PHBV concentration of 0.99 g/l or 0.94 g/l, respectively, as compared to 0.58 g/l in wild type. In the case of PhaC3, the PHBV content (10.02%) and concentration (0.53 g/l) were similar with that of wild type. Compared to that of wild type, the 3HV monomer composition of PHBV was a little higher for PhaEC1 (4.06 mol %), a little lower for PhaEC3 (1.95 mol %), and equivalent for PhaEC_Hme _(3.14 mol %). In short, three types of PHBV with altered 3HV fractions had been synthesized by the *Har. hispanica *PHB-1 recombinants with *phaE*_Hme _and different *phaC *genes.

**Table 2:** PHA accumulation in *Har. hispanica *strains^a^.

*Har. hispanica *Strain	CDW	PHA content	PHA concentration	PHA composition (mol %)	
				
	(g/L)	(wt %)	(g/L)	3HB (C4)	3HV (C5)
Wild type	4.70 ± 0.23	12.26 ± 0.52	0.58 ± 0.03	96.82 ± 0.35	3.18 ± 0.35
PHB-1 (pWL3E)	4.90 ± 0.12	ND	ND	ND	ND
PHB-1 (pWL3EC)	5.27 ± 0.61	17.33 ± 0.04	0.99 ± 0.11	96.86 ± 0.13	3.14 ± 0.13
PHB-1 (pWL3EC1)	6.00 ± 0.07	15.61 ± 0.06	0.94 ± 0.01	95.94 ± 0.50	4.06 ± 0.03
PHB-1 (pWL3EC2)	5.58 ± 0.21	ND	ND	ND	ND
PHB-1 (pWL3EC3)	5.31 ± 0.14	10.02 ± 1.06	0.53 ± 0.07	98.05 ± 0.20	1.95 ± 0.20

^a ^The cells were cultured in MG medium at 37°C for 96 h. For each strain, three samples were cultured and analyzed separately at the same time. Data are shown as the mean ± standard deviation; n = 3. ND, not detectable.

### Properties of PHBV synthesized by different PhaC subunits

A 400 MHz ^1^H NMR study showed that the monomer composition of PHBV produced by the wild-type strain of *Har. hispanica *and its recombinants (data not shown) was in agreement with that determined by the GC analysis (Table 2). Table 3 listed the molecular weights and thermal properties of the accumulated PHBV in the recombinant strains of *Har. hispanica *PHB-1. The number-average molecular weight (*M*_n_) and polydispersity (*M*_w_/*M*_n_) of the polymers were in the range of 22.7-46.5 × 10^4 ^Da and of 1.83-2.16, respectively. The PHBV synthesized by PhaEC3 had the highest M_w _of 91.0 × 10^4 ^Da, two times of that of the PHBV produced by PhaEC1, but the PHBV production was lower (Table 2).

**Table 3:** Molecular weights and thermal properties of PHBV synthesized by three PHA synthases with different PhaC subunits.

**PHBV type**^**a**^	**Molecular weight**^**b**^			**Thermal properties (°C) **^**c**^			
	
	***M***_**w **_**(10**^**4 **^**Da)**	***M***_**n **_**(10**^**4 **^**Da)**	***M***_**w**_**/*M***_**n**_	***T***_**d**_	***T***_**g**_	***T***_**c**_	***T***_**m**_
PhaEC_Hme_-PHBV	34.4	62.8	1.83	252	2.3	54.6	163.2
PhaEC1-PHBV	49.1	22.7	2.16	257	-0.1	50.5	160.3
PhaEC3-PHBV	91.0	46.5	1.96	255	5.7	ND	162.3

^a ^PHBV synthesized by different PHA synthases (PhaEC_Hme_, PhaEC1, or PhaEC3) in *Har. hispanica*; ^b ^*M*_w_, weight-average molecular weight; *M*_n_, number-average molecular weight; ^c ^*T*_d_, temperature at 5% weight loss determined by TGA; *T*_g_, glass-transition temperature; *T*_c_, cold crystallization temperature; *T*_m_, melting temperature; ND, not detectable. The experiments were repeated three times and the results of one representative experiment were shown.

DSC analysis showed that the glass-transition temperatures (*T*_g_) of the PHBV samples were 2.3°C (PhaEC_Hme_-PHBV), -0.1°C (PhaEC1-PHBV), and 5.7°C (PhaEC3-PHBV). This result was consistent with the 3HV monomer composition of the three types of PHBV; a larger amount of 3HV incorporated into PHBV could lead to lower *T*_g_. A similar melting temperature (*T*_m_) was observed for all three PHBV samples, of which the PHBV with the lowest *T*_m _synthesized by PhaEC1 could facilitate its processing ability. Compared with the other two polymers, no cold crystallization temperature (*T*_c_) was detected for the PHBV accumulated by PhaEC3. This result demonstrated that this polymer (with a high 3HB fraction) possessed a fast crystallization speed, which could also provide some advantages in processing.

**Figure 4:**
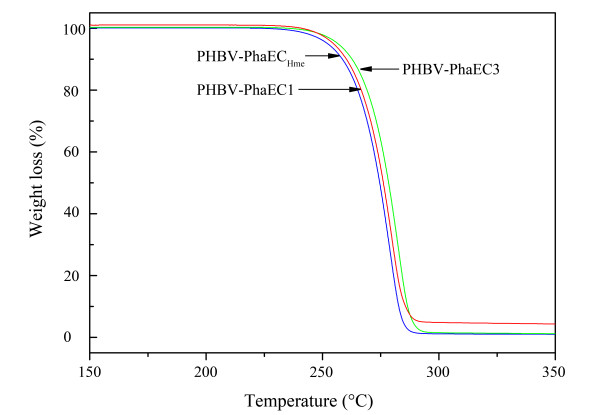
**Thermogravimetric analysis (TGA) of PHBV polymers produced by *Har. hispanica *PHB-1 recombinants harboring PhaEC**_**Hme**_**, PhaEC1, or PhaEC3**.

Thermal stability plays an important role in polymer melt processing. This characteristic of the three types of PHBV was determined by TGA (Table 3 and Figure 4). The temperature at 5% weight loss (*T*_d_) was used to evaluate their polymer thermal stability. The *T*_d _of the three polymers ranged from 252°C to 257°C. Our results showed that the three types of PHBV, with different monomer composition, changed obviously in their thermal properties.

## Discussion

**Figure 5:**
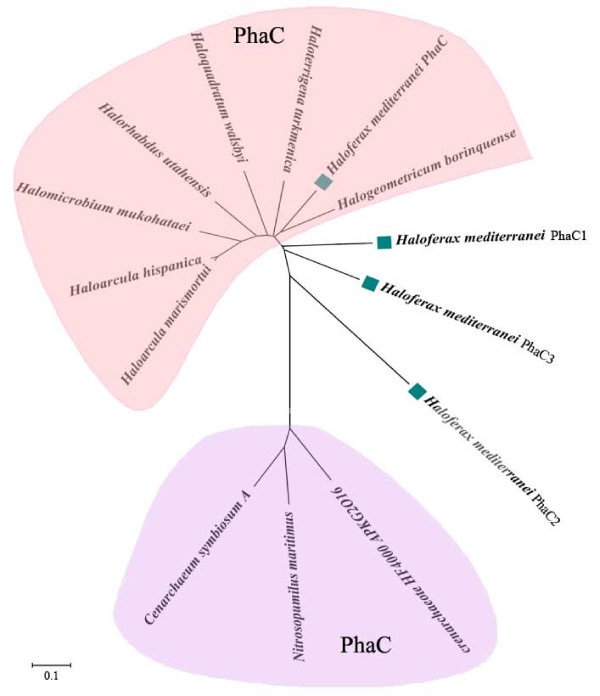
**Phylogenetic analysis of PhaCs from the domain of Archaea**. The tree was constructed using the neighbor-joining algorithm with MEGA software version 4.0. The numbers next to the nodes indicate the bootstrap values based on 1000 replications (expressed as percentages). Scale bar = 0.1 substitution per site. GenBank accession numbers of PhaCs were as follows: *Haloarcula marismortui*, YP_137339; *Hloarcula hispanica*, ABV71394; *Halomicrobium mukohataei*, YP_003176827; *Halorhabdus utahensis*, YP_003131063; *Haloquadratum walsbyi*, YP_658052; *Haloterrigena turkmenica*, YP_003404006; *Halogeometricum borinquense*, ZP_03999959; *Crenarchaeote *HF4000_APKG2016, ABZ08353; *Nitrosopumilus maritimus*, YP_001582325; *Cenarchaeum symbiosum*, YP_876256; *Haloferax mediterranei*, *phaC*_Hme_-EU374220, *phaC1*-HM116246, *phaC2*-HM116247, *phaC3*-HM116248. The four PhaC of *Hfx. mediterranei *are marked with green square.

Multiple *phaC *genes in a same genome have been found in the domain of Bacteria. The complete genome sequence of *Ralstonia eutropha *has revealed the existence of a second *phaC *gene, which is not clustered with the *phaCAB *operon, and its function has not been clarified [25]. Additionally, *Pseudomonas *strains synthesizing medium-chain-length PHAs (mcl-PHAs) usually have two different *phaC *genes, which are separated by a *phaZ *gene that encodes an intracellular PHA depolymerase [1]. To date, the haloarchaeon *Hfx. mediterranei *has been found to possess the largest number of *phaC *genes. Compared with the other PhaC subunits from haloarchaeal strains, the three newly identified PhaCs showed less homology with PhaC_Hme_. A phylogenetic tree was constructed based on the four PhaCs from *Hfx. mediterranei *and seven other PhaCs from *Halobacteriaceae*; 3 PhaC sequences from *Thaumarchaeota *were used as the outgroup (Figure 5). The PhaCs from *Halobacteriaceae *and those from *Thaumarchaeota *clustered separately. PhaC_Hme _was more closely related to the PhaCs from other haloarchaea than to its three paralogs (Figure 5). It seemed likely that *phaC1, phaC2 *and *phaC3 *were not directly acquired from a common source of PhaC_Hme_, but possibly by horizontal DNA transfer from other sources during evolution.

Of the four paralogs in *Hfx. mediterranei*, only *phaC*_Hme _was found to be transcribed under PHA-accumulating conditions by RT-PCR assays (Figure 3A). However, the putative promoters (including the TATA box and BRE element) of the other three *phaC *genes, were also detected (Figure 3B). As both *phaC1 *and *phaC3 *encoded functional proteins, it is conceivable that these silent genes in *Hfx. mediterranei *might be activated under specific ecological conditions to increase the environmental distribution of this haloarchaeon.

Regardless of the conserved catalytic triad residues (Cys-Asp-His) in the primary sequence, PhaC2 was found to have no PHA polymerization activity. This might be the result of the truncated C terminus, which has been shown to be indispensable for PHA synthase enzyme activity [6]. However, a recombinant PhaC2 containing the C terminus from PhaC3 does not have the ability to synthesize PHAs (data not shown). Alternatively, less conserved motifs such as the "Ala" residue in the "Lipase box-like" sequence or the lack of the conserved motif of PhaC might lead to its inactivity.

The incorporation of 3HV into PHB could improve material flexibility. Three types of PHBV with different monomer compositions were produced from the *Har. hispanica *PHB-1 recombinants, and the type of PHBV was supposed to be determined by the different substrate specificities of PhaC_Hme_, PhaC1, and PhaC3. *Hfx. mediterranei *has been found to be the best PHBV producer in the family *Halobacteriaceae*, and can accumulate the highest PHBV content (>60%) with the highest 3HV fraction from many unrelated cheap carbon sources [7,20,21]. Compared with *Har. hispanica*, *Hfx. mediterranei *has a stronger ability to provide more 3HB-CoA and 3HV-CoA monomers for PHA synthase. Therefore, the use of these *phaC *genes in *Hfx. mediterranei *PHBV-1 to produce PHBV with higher 3HV fractions and different properties would have much more promise, which could extend the applications of PHAs in the future.

## Conclusions

In this study, we mined three silent *phaC *genes (*phaC1*, *phaC2*, and *phaC3*) from *Hfx. mediterranei*, which encoded potential PhaCs subunits of PHA synthase. In *Har. hispanica *PHB-1, the heterologous coexpression of *phaE*_Hme _with *phaC*_Hme_, *phaC1*, or *phaC3 *could lead to the accumulation of PHBV polymers with different molecular weights and thermal properties from excess glucose. Therefore, we concluded that genetic engineering of these *phaC *genes in *Hfx. mediterranei *would produce PHBV with desirable material properties, at lower production costs.

## Methods

### Strains, plasmids, and growth conditions

The plasmids used in this study were listed in Table 1. All of the plasmids were derivatives of pWL3E [6]. These plasmids were first constructed in *Escherichia coli *JM109 and then introduced into *Har. hispanica *PHB-1, a *phaEC*-deleted strain [13], using the polyethylene glycol-mediated transformation method [26].

*E. coli *JM109 was cultivated in Luria-Bertani (LB) medium at 37°C [27]. When needed, ampicillin (100 mg/l) was added to the medium. *Har. hispanica *and *Hfx. mediterranei *were grown in AS-168 medium. For growth on plates, the media described above were supplemented with 1.2% (w/v) agar. For PHA accumulation, the haloarchaeal strains were transferred from AS-168 medium to MG medium and cultivated as previously described [6]. When required, mevinolin was added to a final concentration of 5 mg/l for *H. hispanica *recombinants. All of the strains were cultured at 37°C.

### Primers and plasmid construction

All of the primers used in this study were summarized in Table 1. To determine the capabilities of PhaC1, PhaC2, and PhaC3 to synthesize PHAs, three expression plasmids were constructed. The expression vectors were derived from pWL3E, which contained the *phaE*_Hme _gene of *Hfx. mediterranei *with its native promoter [6]. The coding sequences of *phaC1*, *phaC2*, and *phaC3 *were amplified from *Hfx. mediterranei *by PCR with primer pairs phaC1-BamHI-F/phaC1-KpnI-R, phaC2-BamHI-F/phaC2-KpnI-R, and phaC3-BamHI-F/phaC3-KpnI-R, respectively. The purified PCR products (*phaC1*, 1.3 kb; *phaC2*, 1.1 kb; *phaC3*, 1.7 kb) were digested with *Bam*HI and *Kpn*I and inserted into *Bam*HI and *Kpn*I-treated pWL3E. The resulting expression plasmids pWL3EC1, pWL3EC2, and pWL3EC3 were confirmed by DNA sequencing and individually introduced into *Har. hispanica *PHB-1. The haloarchaeal recombinants harboring pWL3EC [6], pWL3EC1, pWL3EC2, and pWL3EC3 were subjected to PHA accumulation analysis in which *Har. hispanica *PHB-1 harboring pWL3E and the wild-type strain were used as the negative and positive control, respectively.

### Isolation of total RNA and reverse transcription PCR (RT-PCR)

Total RNA of *Hfx. mediterranei *was extracted using TRIzol reagent (Invitrogen) from a logarithmic-phase culture grown in MG medium. Four specific primer pairs, phaC-RT-F/phaC-RT-F, phaC1-RT-F/phaC1-RT-F, phaC2-RT-F/phaC2-RT-F, and phaC3-RT-F/phaC3-RT-F, were designed for the *phaC*_Hme_, *phaC1*, *phaC2*, and *phaC3 *genes, respectively (Table 1). Prior to RT-PCR, the specificity of these primers were confirmed by sequencing of the corresponding PCR products using the genomic DNA of *Hfx. mediterranei *as template. For RT-PCR, the RNA sample was digested with RNase-free RQ1 DNase (Promega) overnight to eliminate any DNA contamination, which was subsequently confirmed by a control PCR without prior reverse transcription as previously described [28]. The DNA-free RNA sample was then used as the template for RT-PCR using the OneStep RT-PCR Kit (Qiagen) according to the manufacturer's instruction. The PCR products were electrophoresed in a 1% agarose gel.

### Measurement of PHA content and composition

Scl-PHAs were qualitatively and quantitatively analyzed by gas chromatography (GC) with the Agilent GC-6820 as described previously [6,13]. Benzonic acid was used as an internal standard to calculate the amount of PHAs. PHBV (Sigma) was used as the standard for peak identification. The average results of three parallel experiments were recorded.

### Extraction and purification of PHAs

After fermentation, the liquid cultures were harvested by centrifugation at 9000 rpm for 10 min. The cell pellets were resuspended in distilled water containing 0.1% SDS (w/v) and agitated for 20 min. The suspension was centrifuged at 5000 rpm for 5 min and yielded whitish, compact sediment. The sediment was resuspended and washed twice with 0.1% SDS solution (w/v) and distilled water, respectively. The resulting paste was dried in an oven at 70°C for 2 h for further purification. The extracted PHAs were treated with hot chloroform at 90°C for 4 h, followed by precipitation with 15 volume of pre-chilled ethanol. To obtain pure product, the precipitate was then redissolved in chloroform and the process was repeated. The collected PHAs were dried at 40°C for 24 h to remove all residual solvent.

### Molecular weight determination of PHBV

The molecular weight of PHBV was estimated *via *gel permeation chromatography (GPC) with a Waters 1515 system at 30°C using a Waters 1525 pump equipped with a four Styragel column series (Styragel HR, 5 μm). A differential refractive index detector and a UV detector were both employed. Chloroform was used as the elution solvent at a flow rate of 1.0 ml/min. The concentration of the samples was 2 mg/ml and the injection volume was 50 μl. The calibration curve was generated with polystyrene standards (Sigma).

### Characterization of PHBV thermal properties

Differential scanning calorimetry (DSC) was performed using a TA Instruments Q100 with an autocool accessory calibrated with indium to characterize thermal transitions of the polymers. The following protocol was used for each sample. A 3-5 mg sample in an aluminum pan was cooled from room temperature to -60°C by the autocool accessory. The pan was heated from -60°C to 180°C at a rate of 10°C/min, isothermally maintained at 180°C for 5 min, quenched to -60°C, and reheated from -60°C to 180°C at 10°C/min under a nitrogen flow rate of 50 ml/min. Data were collected during the second heating run.

Thermal stability of the PHA polymer was determined *via *thermogravimetric analysis using a Q50 thermogravimetric analyzer (TGA) (TA Instruments). The procedures used were as the same as those described by Xie *et al. *[29]. Briefly, samples were analyzed at a heating rate of 10°C/min under a nitrogen atmosphere with a N2 flow rate of 60 ml/min.

### Phylogenetic tree construction

The phylogenetic tree for PhaC was constructed using the neighbor-joining method [30] with Molecular Evolutionary Genetics Analysis (MEGA) software version 4.0 [31]. The topology of the phylogenetic tree was evaluated by bootstrap analysis on the basis of 1000 replications [32].

### Nucleotide and protein sequence analysis

Sequences of the PhaC proteins from *Hfx. mediterranei *were deposited in GenBank under the accession numbers *phaC*_Hme_-EU374220, *phaC1*-HM116246, *phaC2*-HM116247, and *phaC3*-HM116248. These PhaC sequences were analyzed using the GeneDoc program (http://www.nrbsc.org/gfx/genedoc/index.html).

## Competing interests

The authors declare that they have no competing interests.

## Authors' contributions

JH (Han) performed most of the experimental work and drafted the manuscript. ML extracted the total RNA and was involved in gene expression analysis. JH (Hou) and JZ extracted and purified the polymers. LW was involved in copolymer characterization. HX was involved in experimental design, data analysis, and finalized the manuscript. All the authors have read and approved the final manuscript.
